# Maturation-Dependent Licensing of Naive T Cells for Rapid TNF Production

**DOI:** 10.1371/journal.pone.0015038

**Published:** 2010-11-24

**Authors:** Bhavana Priyadharshini, Raymond M. Welsh, Dale L. Greiner, Rachel M. Gerstein, Michael A. Brehm

**Affiliations:** 1 Department of Pathology, University of Massachusetts Medical School, Worcester, Massachusetts, United States of America; 2 Program in Immunology and Virology, University of Massachusetts Medical School, Worcester, Massachusetts, United States of America; 3 Program in Molecular Medicine, University of Massachusetts Medical School, Worcester, Massachusetts, United States of America; 4 Diabetes Center of Excellence, University of Massachusetts Medical School, Worcester, Massachusetts, United States of America; 5 Department of Molecular Genetics & Microbiology, University of Massachusetts Medical School, Worcester, Massachusetts, United States of America; Centre de Recherche Public de la Santé (CRP-Santé), Luxembourg

## Abstract

The peripheral naïve T cell pool is comprised of a heterogeneous population of cells at various stages of development, which is a process that begins in the thymus and is completed after a post-thymic maturation phase in the periphery. One hallmark of naïve T cells in secondary lymphoid organs is their unique ability to produce TNF rapidly after activation and prior to acquiring other effector functions. To determine how maturation influences the licensing of naïve T cells to produce TNF, we compared cytokine profiles of CD4^+^ and CD8^+^ single positive (SP) thymocytes, recent thymic emigrants (RTEs) and mature-naïve (MN) T cells during TCR activation. SP thymocytes exhibited a poor ability to produce TNF when compared to splenic T cells despite expressing similar TCR levels and possessing comparable activation kinetics (upregulation of CD25 and CD69). Provision of optimal antigen presenting cells from the spleen did not fully enable SP thymocytes to produce TNF, suggesting an intrinsic defect in their ability to produce TNF efficiently. Using a thymocyte adoptive transfer model, we demonstrate that the ability of T cells to produce TNF increases progressively with time in the periphery as a function of their maturation state. RTEs that were identified in NG-BAC transgenic mice by the expression of GFP showed a significantly enhanced ability to express TNF relative to SP thymocytes but not to the extent of fully MN T cells. Together, these findings suggest that TNF expression by naïve T cells is regulated via a gradual licensing process that requires functional maturation in peripheral lymphoid organs.

## Introduction

T cell development begins within the thymus and is driven to completion after single positive (SP) thymocytes exit the thymus and seed secondary lymphoid organs, where they undergo progressive phenotypic and functional maturation [1]. The peripheral naïve T cell pool is therefore comprised of a heterogeneous population of cells at different stages of post-thymic development, encompassing T cell subsets from the fully mature to the most recently emigrated thymic T cells [2]. The recent thymic emigrants (RTEs), which are 0–2 weeks old in the periphery have a distinct phenotypic profile (CD24^high^, Qa2^low^, CD45RB^low^) relative to their mature naïve (MN) counterparts, that are resident in the periphery for >3 weeks (CD24^low^, Qa2^high^, CD45RB^high^) [1], [3]. RTEs have been shown to also differ functionally, producing less IL-2, exhibiting a decreased ability to proliferate upon 48 hours of in vitro TCR stimulation and producing less IFN-γ after 7 days of infection with ovalbumin-expressing *Listeria monocytogenes* (*r*LM-OVA) [1], [3]. Resting naïve T cells in secondary lymphoid organs are quiescent in nature requiring a low level of TCR signaling from self peptide-MHC ligands to maintain immune homeostasis [4]. Upon antigen-specific activation, naïve T cells differentiate and clonally expand to become effectors that are capable of secreting cytokines (IL-2, TNF and IFN-γ) and exhibiting cytolytic function [5], [6], [7]. In contrast to this conventional paradigm, naïve CD4^+^ and CD8^+^ T cells (CD44^lo^, CD11a^lo^) have recently been shown to rapidly produce TNF within 4 to 5 hours of TCR engagement, before ensuing cell division or producing other effector cytokines such as IL-2 or IFN-γ [8], [9]. The kinetics of TNF production by naïve T cells suggest that this potent immunomodulatory cytokine is released during the initial encounter between T cells and APCs, a critical phase in the programming of antigen-specific responses [5], [10], [11], [12], [13], [14], [15], [16]. However, when and how naïve T cells acquire this unique capability to produce TNF during development is not known.

TNF is a potent pro-inflammatory cytokine that elicits pleiotropic effects during an immune response, affecting immune cell activation, survival, death and differentiation [17], [18]. The effects of TNF are mediated through two distinct receptors, TNFR1 (p55) and TNFR2 (p75) [19], [20], [21], [22]. Deregulation of TNF signaling pathways has been implicated in the pathogenesis of several diseases, including rheumatoid arthritis (RA), Crohn's disease (CD), inflammatory bowel disease (IBD) and multiple sclerosis (MS), and hence therapeutic agents that target and block the activity of TNF have been developed for clinical use [21], [23], [24], [25], [26], [27], [28]. In addition to being a major inducer of inflammation during innate immune responses, TNF signaling also mediates immunomodulatory effects in adaptive immune responses [29]. For example, TNF signaling plays a vital role in the generation of functional T cell responses to tumor antigens, DNA vaccines and recombinant adenoviruses [23], [30], [31], [32]. More specifically, signaling through TNFR2 but not TNFR1 has a synergistic role with CD28 co-stimulation, reducing the threshold of activation for optimal IL-2 expression during the initial stages of T cell activation [23], [33], [34], [35]. In contrast, there is evidence suggesting a suppressive role for TNF in the generation of T cell responses after infection of mice with LCMV. For example, higher frequencies of LCMV-specific CD4^+^ and CD8^+^ memory T cells are detectable in mice with defective TNF signaling pathways [36], [37], [38], [39]. These studies together indicate that effects of TNF signaling on the induction of adaptive immune responses are dependent on the nature of the antigenic challenge.

Given the important role of TNF in regulating immune responses, here we determined the developmental stage when naïve T cells become competent to produce TNF by comparing the capability of SP naïve T cells to produce TNF before and after emigration from the thymus. These studies reveal that CD4^+^ CD8^−^ and CD4^−^ CD8^+^ SP thymocytes possess a poor ability to produce TNF upon stimulation when compared to their counterparts in secondary lymphoid organs. Contact with secondary lymphoid cells (spleen and lymph node) during TCR activation partially enables SP thymocytes to produce TNF in vitro by providing optimal antigen-presentation. However, the frequency of TNF producing cells is still significantly lower than in the periphery. RTEs in the spleen on the other hand, display an intermediate TNF response, which is higher than their SP thymic precursors but lower relative to the fully MN T cells. The differences in the TNF profile exhibited by these 3 populations of lymphocytes mirrors their distinctive maturation status. Moreover, as developing T cells mature in the periphery, they show a progressive increase in their capability to produce TNF upon TCR activation. Together, these findings suggest that naïve T cells become gradually licensed to efficiently produce TNF in a maturation-dependent manner that requires their localization to secondary lymphoid organs.

## Results

### SP thymocytes have an impaired ability to produce TNF after TCR activation

**Figure 1:**
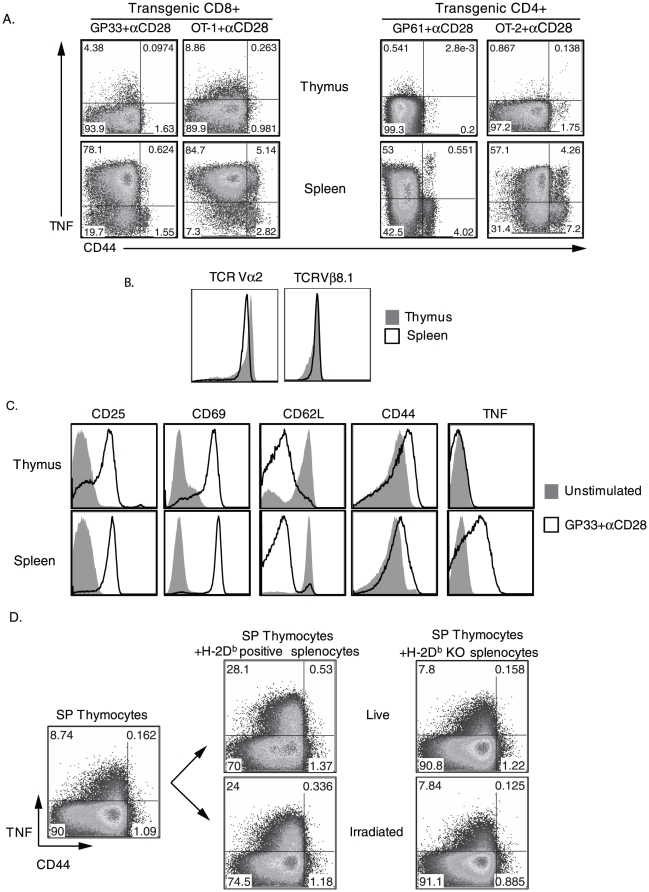
SP thymocytes are poor TNF producers relative to naïve splenic T cells during TCR activation in vitro. Thymocytes and splenocytes from P14-CD8^+^, OT-1-CD8^+^, SMARTA-CD4^+^ and OT-2-CD4^+^ TCR transgenic mice were stimulated in vitro as indicated for 4 hours and then stained for intracellular TNF cytokine, as described in *Materials and Methods*. For analysis, the cells were gated on either SP CD8^+^ CD4^−^ or SP CD4^+^ CD8^−^ cells. A, The percentages of TCR transgenic thymocytes and splenic T cells (both CD44^lo^ and CD44^hi^) staining positive for TNF are shown. B, Resting P14-CD8^+^ thymocytes and naïve splenocytes were stained with mAbs to Vα2 and Vβ8.1. The profile for the SP thymocytes is shown in gray solid histograms and for splenic T cells in black line histograms. C, P14-CD8^+^ thymocytes and naïve splenocytes were either unstimulated (gray solid histograms) or stimulated (black line histograms) for 4 hours with GP33+αCD28 in vitro and stained with mAbs to the indicated surface molecules. D, CD45.1^+^ SP P14-CD8^+^ thymocytes were enriched and stimulated with GP33+αCD28 for 4 hours either alone or in the presence of live or irradiated (3000cGy) H-2D^b^ WT or H-2D^b^ KO splenocytes respectively. Cells were then stained for intracellular TNF. The percentages of CD45.1^+^ CD8^+^ thymocytes (CD44^lo^ and CD44^hi^) staining positive for TNF are shown.

Naïve CD4^+^ and CD8^+^ T lymphocytes (CD44^lo^) from secondary lymphoid organs rapidly produce TNF after TCR engagement before gaining other effector functions [8]. However, it is not known at what stage of development naïve T cells acquire the ability to produce TNF. To determine this, thymocytes and splenocytes from CD8^+^ and CD4^+^ TCR-transgenic mice (P14, OT-1 & SMARTA and OT-2) were stimulated with specific peptides and αCD28 costimulation for 4 hrs in vitro. Fig. 1A shows that a lower proportion of CD4^+^ CD8^−^ and CD4^−^ CD8^+^ SP thymocytes produced TNF when compared to naïve (CD44^lo^) splenic T cells during TCR stimulation. This inability to produce TNF was not overcome by increasing the concentrations of the peptide (data not shown). To determine if the reduced TNF response by SP thymocytes was due to a lower TCR expression on SP thymocytes relative to naïve splenic T cells [40], [41], [42], SP P14-CD8^+^ thymocytes and naïve (CD44^lo^) splenic T cells were stained with mAbs to TCR Vα2 and TCR Vβ8.1. Fig. 1B shows that Vα2 and Vβ8.1 expression in SP P14-CD8^+^ thymocytes and splenic T cells were similar. Next, to determine if the reduced ability of SP thymocytes to produce TNF was due to a generalized defect in their activation, we examined the expression of activation markers CD25, CD69, CD44 and CD62L on the P14-CD8^+^ SP thymocytes and splenic T cells. As shown in Fig. 1C, SP thymocytes and splenic T cells exhibited a comparable level of activation at 4 hours, with the expression of CD25 and CD69 being up-regulated and the expression of CD62L down-regulated, as previously shown [8], [43]. These results suggest that SP thymocytes are incompetent to produce TNF when compared to splenic T cells upon TCR stimulation despite exhibiting similar TCR levels and similar phenotypic changes in the expression of activation markers.

### Optimal antigen presentation is not sufficient to enable thymocytes to produce TNF efficiently

Recent studies have shown that thymic DCs and splenic DCs have unique properties and that the microenvironment contributes to their distinct functions [44]. We hypothesized that differences in TNF production between SP thymocytes and naïve splenic T cells may be attributed to the differences in antigen presentation between the two organs. To test this, enriched CD45.1^+^ SP P14-CD8^+^ thymocytes were stimulated in the presence of either WT CD45.2^+^ H-2D^b^-positive B6 splenocytes or CD45.2^+^ H-2D^b^-deficient B6 splenocytes (incapable of presenting GP33 to P14 cells). In comparison to the high frequencies of TNF-producing splenocytes depicted in Fig. 1A, there was only a partial increase in the proportion of SP P14-CD8^+^ thymocytes that produced TNF, when stimulated in the presence of WT B6 splenocytes (Fig. 1D). Both purified splenic B cells and non-B and -T cell populations that contained CD11c^+^ APCs in the flow-through stimulated this small increase in TNF production (21.4% and 17.3% of SP P14 CD8^+^ thymocytes were TNF positive when stimulated purified B cells and non-B and -T cell populations, respectively). We then determined whether SP P14-CD8^+^ thymocytes produced TNF as a consequence of changes occurring in antigen presenting cells during the co-culture, by using irradiated splenocytes. We found that SP P14-CD8^+^ thymocytes stimulated with irradiated (3000cGy) WT B6 splenocytes showed a similar increase in the proportion of TNF producing cells comparable to SP P14-CD8^+^ thymocytes stimulated with live WT B6 splenocytes (Fig. 1D), indicating that viable splenocytes were not necessary for this effect. As expected, thymocytes stimulated in the presence of H2D^b^-deficient splenocytes (live or irradiated) did not produce TNF. Together, these results indicate that although optimal TCR-MHC interactions provided by spleen cells enable a small subset of SP thymocytes to produce TNF, the frequency of cells and the level of TNF produced on a per cell basis was reduced compared to peripheral naïve CD8^+^ splenic T cells, suggesting an intrinsic defect in SP thymocytes to produce TNF efficiently upon TCR activation.

### Lower level of TNF transcription in SP thymocytes relative to naïve splenic T cells during TCR activation

**Figure 2:**
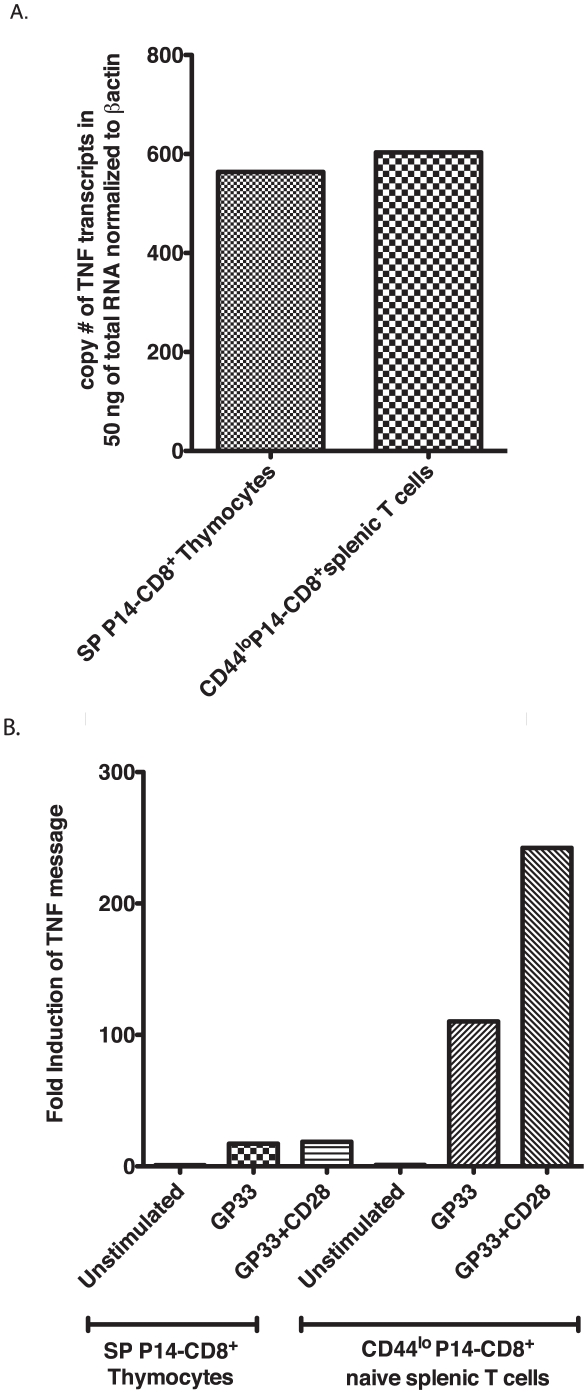
Reduced upregulation of TNF message in SP thymocytes relative to naïve splenic T cells upon TCR stimulation. SP P14-CD8^+^ thymocytes and (CD44^lo^) naïve splenic CD8^+^ T cells were purified by cell sorting and stimulated in the presence of GP33 and GP33+αCD28 for 4 hours followed by RNA isolation and cDNA synthesis from 50 ng of RNA and then amplified using TNF specific primers by quantitative real time PCR from the indicated populations as described in *Materials and Methods*. A, The basal steady state level of TNF transcripts in 50 ng of total RNA (normalized to a β-actin control) isolated from unstimulated SP P14-CD8^+^ thymocytes and (CD44^lo^) naïve splenic T cells are shown. B, The increase in TNF message in these subsets upon stimulation with GP33 and GP33+αCD28 in terms of fold induction with respect to unstimulated SP thymocytes been (normalized to a β-actin control) are shown. This profile is representative of 3 individual experiments.

We wanted to determine if the reduced ability of thymocytes to produce TNF may be related to the levels of mature TNF message expressed within SP thymocytes. Therefore, we quantified the steady-state levels of mature TNF mRNA in SP thymocytes CD4^−^CD8^+^ and their respective naïve splenic (CD44^lo^) counterparts purified from (transgenic) P14 mice at resting state. SP P14-CD8^+^ thymocytes were sorted to 98% purity and (CD44^lo^) P14-CD8^+^ splenic T cells were sorted to 90.3% purity Fig. 2A shows the copy number of TNF transcripts detected in the indicated groups by quantitative real-time PCR. The levels of mature TNF message in transgenic SP thymocytes (CD4^−^CD8^+^) and their naïve splenic counterparts were similar and the differences were not significant (Fig. 2A). Next we compared the levels of TNF transcripts in purified SP P14-CD8^+^ thymocytes and naïve (CD44^lo^) splenic T cells that were stimulated as indicated. We found that the levels of TNF mRNA were dramatically higher in (CD44^lo^) P14-CD8^+^ splenic T cells during GP33 and GP33+αCD28 stimulation relative to SP thymocytes. The levels of TNF transcripts increased in the thymic subsets upon stimulation but not to the extent detected in the splenic subset (Fig. 2B). Together, these results indicate that despite having a basal level of transcription of the TNF gene, SP thymocytes appear to lack the ability to induce TNF transcription efficiently upon stimulation relative to naïve splenic T cells.

### Differential ability of SP thymocytes and naïve splenic T cells to produce TNF during TCR activation *in vivo*

**Figure 3:**
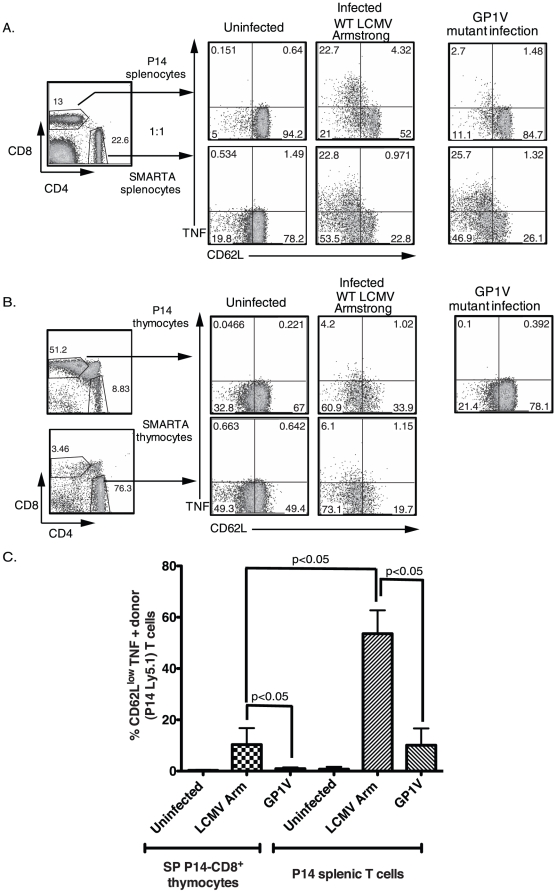
SP thymocytes and splenic T cells exhibit a differential ability to produce TNF during in vivo TCR activation. A and B, CD45.1^+^ P14-CD8^+^ and SMARTA-CD4^+^ splenocytes were mixed at 1∶1 ratio, treated with bfA and transferred into CD45.2^+^ congenic hosts that were uninfected or infected with either WT LCMV Armstrong or the LCMV variant GP1V. CD45.1^+^ P14-CD8^+^ and SMARTA-CD4^+^ thymocytes were treated with bfA and transferred separately into CD45.2^+^ congenic hosts that were infected with either WT LCMV Armstrong or the viral variant GP1V. Spleens from all recipient mice were harvested 4 hours later and directly stained for intracellular TNF. The percentages of donor CD45.1^+^ P14-CD8^+^ and SMARTA-CD4^+^ splenic T cells and SP thymocytes that are TNF positive and have down-regulated CD62L are shown. C, The average percentages of CD62L^lo^TNF^+^ donor T cells detected ex vivo are shown. One-way ANOVA with Tukey post-test was used to compare the mice that received P14 thymocytes or splenocytes and were either uninfected or infected with WT LCMV strain or the viral mutant strain (p<0.05). These data are a pool of 6 experiments. Error bars indicate SD.

To first determine if naïve splenic T cells produced TNF in the presence of physiologically relevant levels of antigen, we performed an in vivo cytokine assay [45]. Briefly, CD45.1^+^ P14-CD8^+^ and CD45.1^+^ SMARTA-CD4^+^ TCR-transgenic splenic T cells were treated in vitro with bfA, a golgi transport inhibitor that blocks cytokine secretion. These cells were then mixed and co-transferred into recipients that were infected with either WT LCMV-Armstrong or a GP33-CTL escape variant of LCMV (GP1V) for 2 days. The spleens of recipient mice were recovered 4 hours later and directly stained for intracellular TNF by the donor T cells. Both P14-CD8^+^ and SMARTA-CD4^+^ donor T cells produced TNF in mice that were infected with WT LCMV Armstrong (Fig. 3A). Only T cells that had down-regulated CD62L, which is consistent with a TCR-mediated activation event, were able to produce TNF. In contrast, P14 -CD8^+^ donor T cells in mice that were infected with GP1V mutant virus were impaired in their ability to produce TNF (Fig. 3A) while SMARTA-CD4^+^ donor T cells were unaffected. These findings indicate that the in vivo production of TNF by naïve CD8^+^ T cells during LCMV infection was specifically initiated by TCR-mediated signaling and was not due to non-specific effects on naïve T cells by virus-induced inflammation.

We next examined the ability of SP P14-CD8^+^ thymocytes and SMARTA-CD4^+^ thymocytes to produce TNF in the same scenario. A small but reproducible proportion of both donor SP P14-CD8^+^ and SMARTA-CD4^+^ thymocytes produced TNF (4 to 6% TNF positive of total T cells) during LCMV infection (Fig. 3B). As expected, infection with GP1V mutant impaired the ability of the donor SP P14-CD8^+^ thymocytes to produce TNF. Fig. 3C shows the average percentages of TNF producing SP P14-CD8^+^ donor thymocytes and splenic T cells that had downregulated their CD62L expression under the indicated conditions. Together, these results confirm our in vitro data indicating that SP thymocytes are impaired in their ability to produce TNF efficiently when compared to naïve splenic T cells during a viral infection.

### TNF producing capability of SP thymocytes correlates with their maturation state

**Figure 4:**
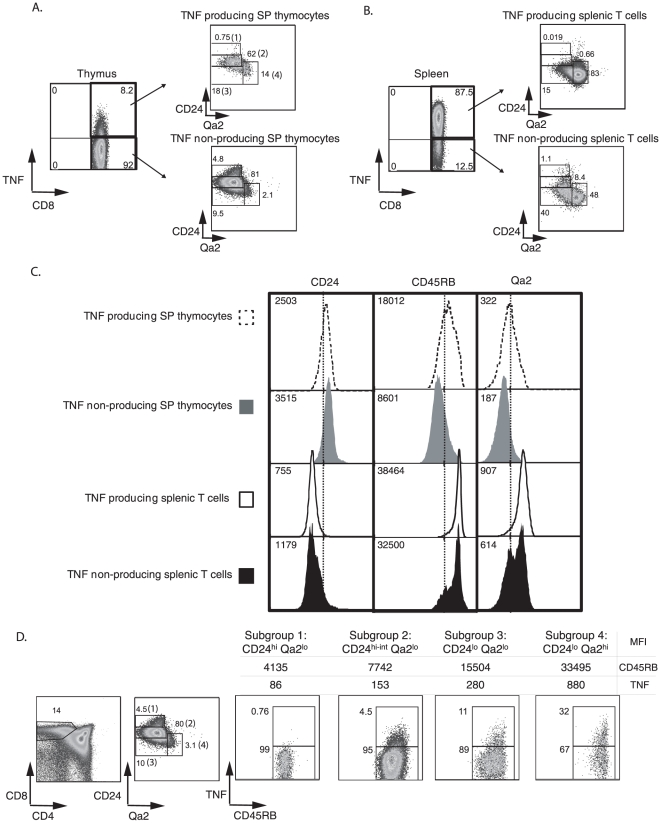
TNF producing SP thymocytes exhibit a lower maturation profile relative to their splenic counterparts. A and B, CD45.1^+^ P14-CD8^+^ thymocytes were stimulated with GP33+αCD28 for 4 hours in vitro and then stained for maturation markers and intracellular TNF, as described in *Materials and Methods*. The TNF producers and non producers of the thymic and the splenic subsets were each classified into 4 subgroups based on their CD24 and Qa2 expression as shown namely Subgroup 1 (CD24^hi^ Qa2^lo^) followed by Subgroup 2 (CD24^hi-int^ Qa2^lo^), Subgroup 3 (CD24^lo^ Qa2^lo^) and finally Subgroup 4 (CD24^lo^ Qa2^hi^). C, shows the histogram comparison of the small population of TNF producing SP thymocytes (dotted line histograms), the majority of SP thymocytes that are TNF non-producers (gray histograms) and TNF producing splenic T cells (solid dark line histograms) and TNF non-producing splenic T cells (black histograms). The MFIs of each of the maturation markers in TNF producing and non-producing thymic and splenic T cells are indicated in the left hand side of the histograms respectively. D, shows the maturation profile of the total SP CD8^+^ thymocytes based on their CD24 and Qa2 expression. The proportion of cells capable of making TNF in the 4 subgroups is shown with respect to CD45RB expression.

SP thymocytes are comprised of a heterogeneous population consisting of cells at different levels of maturity [46]. Immature SP thymocytes express high levels of CD24 (HSA), which is down-regulated as cells progress into maturity [47]. This is accompanied by down-regulation of CD69 and the up-regulation of other markers such as CD62L, CD45RB and Qa2 [48], [49], [50]. We first compared the maturation profile of the total TNF-producing thymocytes with their TNF non-producing counterparts. We broadly classified SP P14-CD8^+^ thymocytes based on their maturation status determined by CD24 and Qa2 expression. CD8 SP thymocytes were divided into 4 subgroups from the least mature to most mature (Fig 4A). Subgroup 1 was comprised of CD24^hi^ Qa2^lo^ cells (least mature) followed by subgroup 2 (CD24^hi-int^Qa2^lo^), subgroup 3 (CD24^lo^ Qa2^lo^) and finally subgroup 4 (CD24^lo^ Qa2^hi^) which was the most mature [51]. The small population of SP P14-CD8^+^ thymocytes that produced TNF displayed a more mature phenotypic profile with the majority of the TNF producers falling in subgroups 2 and 3 compared to the TNF non-producers that fell mostly in subgroups 1 and 2. The maturation differences between the TNF-producing SP thymocytes and the non-producers were also seen in the MFI changes in CD24, CD45RB and Qa2 (dotted line histograms and gray histograms in Fig 4C). However, the TNF producing SP P14-CD8^+^ thymocytes had a less mature phenotype when compared to their splenic counterparts. As described in Fig 4A and Fig 4B, >60% of the TNF producing thymocytes constituted subgroups 2 and 3 relative to the TNF producing splenic T cells that constituted >80% in subgroups 3 and 4. The differences were also reflected in the MFI of maturation markers (dark line histograms and black histograms in Fig. 4*D*). We next examined the TNF-producing capability of each of the 4 subgroups in the SP thymic subset individually. The subgroups showed increasing mean fluorescence intensities of CD45RB, consistent with their maturation state (Fig 4D). There was a progressive increase in TNF production on a per cell basis that correlated with maturation with Subgroup 4 having the highest percentage of TNF^+^ cells. Together, these results suggest that though the small population of TNF producing SP P14-CD8^+^ thymocytes is more mature than the TNF non-producing counterparts, these cells are still phenotypically less mature than P14-CD8^+^ naïve T cells localized in the spleen.

### Adoptively transferred transgenic SP thymocytes progressively gain the ability to produce TNF in the periphery

**Figure 5:**
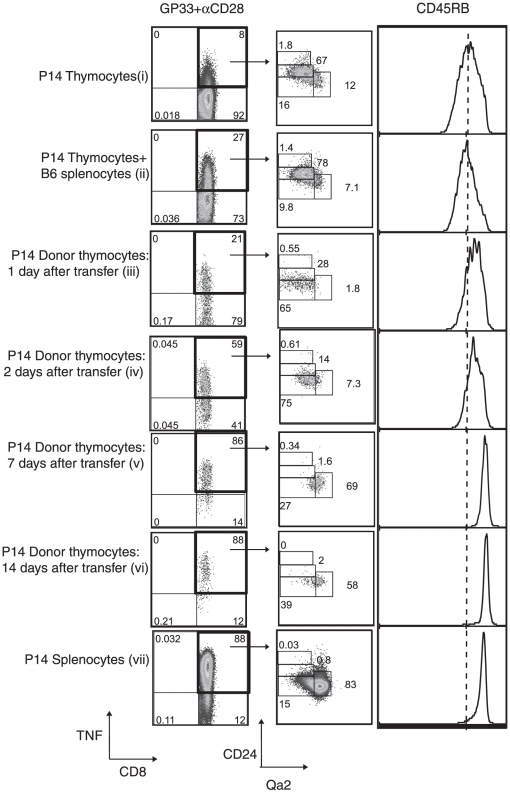
Post-thymic maturation status of naïve P14 transgenic T cells determines their TNF producing capability. Female CD45.1^+^ P14-CD8^+^ thymocytes were transferred into female CD45.2^+^ B6 congenic mice. Host spleens were recovered after the indicated time periods and were stimulated in vitro for 4 hours with GP33+αCD28 and donor CD45.1^+^ T cells were stained for intracellular TNF as described in *Materials and Methods*. Dead cells were excluded using Live Dead Aqua Dead cell stain for this experiment. For analysis, cells were gated on the live donor SP P14-CD8^+^ T cells and the maturation profile of donor cells that are TNF^+^ (indicated by arrows in the boxed quadrants in plots iii,iv,v,vi) were compared at all the time points shown (corresponding histograms). Additionally, some CD45.1^+^ P14-CD8^+^ thymocytes were stimulated before transfer in the context of CD45.2^+^ B6 splenocytes in vitro for 4 hours with GP33+αCD28 and their maturation profile was compared to CD45.1^+^ P14-CD8^+^ thymocytes and splenocytes stimulated alone in vitro (plots i, ii and vii).

The differences in TNF production between SP P14-CD8^+^ thymocytes and naïve P14-CD8^+^ splenic T cells upon TCR stimulation parallels the differences in the maturation status of T cells in these two compartments as shown in Fig 4. The functional maturation of developing T cells occurs progressively with time upon contact with secondary lymphoid organs after their exit from the thymus [1]. Given this, we hypothesized that SP thymocytes migrating into the periphery will gradually acquire the capability to produce TNF efficiently upon TCR stimulation. To recapitulate thymic emigration, 20×10^6^ CD45.1^+^ P14 thymocytes were adoptively transferred into uninfected CD45.2^+^ B6 congenic mice. Spleens were harvested from recipient mice at the indicated time points (Fig 5: plots iii,iv,v,vi) and stained for donor CD45.1^+^ SP P14-CD8^+^ thymocytes producing TNF upon in vitro TCR stimulation. The proportion of donor CD45.1^+^ SP P14-CD8^+^ thymocytes producing TNF upon TCR stimulation increased over the time of the experiment (boxed quadrants in Fig 5: plots iii,iv,v,vi). The donor CD45.1^+^ SP P14-CD8^+^ thymocytes capable of TNF production also exhibited an increasing maturation phenotype (down-regulation of CD24 and up-regulation of CD45RB and Qa2) that approached a level similar to that of splenic T cells by day 14 after transfer. While the recovery of donor cells diminished over time, as shown in Table 1, we also observed increases in the mean fluorescence intensity (MFI) of the TNF signal in naïve (CD44^lo^) donor CD45.1^+^ SP P14-CD8^+^ thymocytes producing TNF from day 2 to day 14 after transfer (Table 1). This increase in expression of TNF on a per cell basis by donor SP CD45.1^+^ P14-CD8^+^ thymocytes was significant (p<0.05) and was consistent with the increasing maturation phenotype observed at these time points (Fig 5 and Table 2). We next compared the changes in MFI of maturation markers in the TNF-producing and non-producing donor thymocytes at day 1 and 2 after transfer, as the TNF ^negative^ populations were very small at later time points (Table 2). The TNF-producing cells were more mature, again suggesting that the changes in the maturation state of donor thymocytes correlated with increasing capability to produce TNF efficiently on a per cell basis. Pre-transfer stimulation of thymocytes ex vivo in the presence of CD45.2^+^ B6 splenocytes did not affect their maturation status. Together, these results suggest that the progressive maturation of transferred SP P14-CD8^+^ thymocytes in the periphery positively influences their capability to competently produce TNF upon TCR stimulation.

**Table 1:** SP thymocytes acquire the ability to produce TNF as a function of time in the periphery^a^.

Group	Days post-transfer	Absolute number of donor P14-CD8^+^ T cells (×10^4^)	TNF MFI of TNF^+^CD44^lo^ donor T cells (×10^2^)
1.	Day 1	48±8	24±4
2.	Day 2	36±18	24±2
3.	Day 7	14±10^b^	47±4^d^^,^ f
4.	Day 14	5±2^c^	64±19^e^^,^ g ^,^ h

a The recovery of the donor (CD44^lo^) SP P14-CD8^+^ thymocytes from recipient spleens and the MFI of TNF expression at the indicated time points post-transfer are shown. The average recovery and the MFI of TNF expression by donor thymocytes (n = 6 per time point) were analysed using One-way ANOVA with a Tukey post-test as described in *Materials and Methods*. Error indicates SD. N/A, Not Applicable.

b p<0.05 vs Table 1, group 1.

c p<0.05 vs Table 1, group 1.

d p<0.05 vs Table 1, group 1.

e p<0.05 vs Table 1, group 1.

f p<0.05 vs Table 1, group 2.

g p<0.05 vs Table 1, group 2.

h p<0.05 vs Table 1, group 3.

**Table 2:** Maturation state of SP thymocytes reflects their TNF producing capability^a^.

Group	Days post-transfer	CD24 MFI of donor T cells (×10^2^)		CD45RB MFI of donor T cells (×10^2^)		Qa2 MFI of donor T cells (×10^2^)	
		TNF+	TNF−	TNF+	TNF−	TNF+	TNF−
1.	Day1	24±6	34±7^h^	239±15	137±14^j^	1.4±0.1	0.1±0.1
2.	Day2	20±3	31±5^i^	305±33^d^	244±37	4.9±2.2	2.8±0.8
3.	Day7	15±3^b^	N/A	588±26^e^	N/A	7.4±0.8	N/A
4.	Day14	13±2^c^	N/A	629±27^f^	N/A	11.5±5.6^g^	N/A

a The average MFI of maturation markers CD24 (n = 6), CD45RB (n = 6) and Qa2 (n = 3 for group1 and 2 and n = 6 for for group 3 and 4) in TNF producing donor SP P14-CD8^+^ are shown. The averages were analysed using One-way ANOVA with a Tukey post-test as described in *Materials and Methods*. Error indicates SD. N/A, Not Applicable.

b p<0.05 vs Table II, group 1.

c p<0.05 vs Table II, group 1.

d p<0.05 vs Table II, group 1.

e p<0.05 vs Table II, group 1.

f p<0.05 vs Table II, group 1.

g p<0.05 vs Table II, group 1.

h p<0.05 vs Table II, group 1 TNF+.

i p<0.05 vs Table II, group 1 TNF+.

j p<0.05 vs Table II, group 1 TNF+.

### Post-thymic maturation of naturally emigrating polyclonal SP thymocytes licenses them to produce TNF efficiently in the periphery

**Figure 6:**
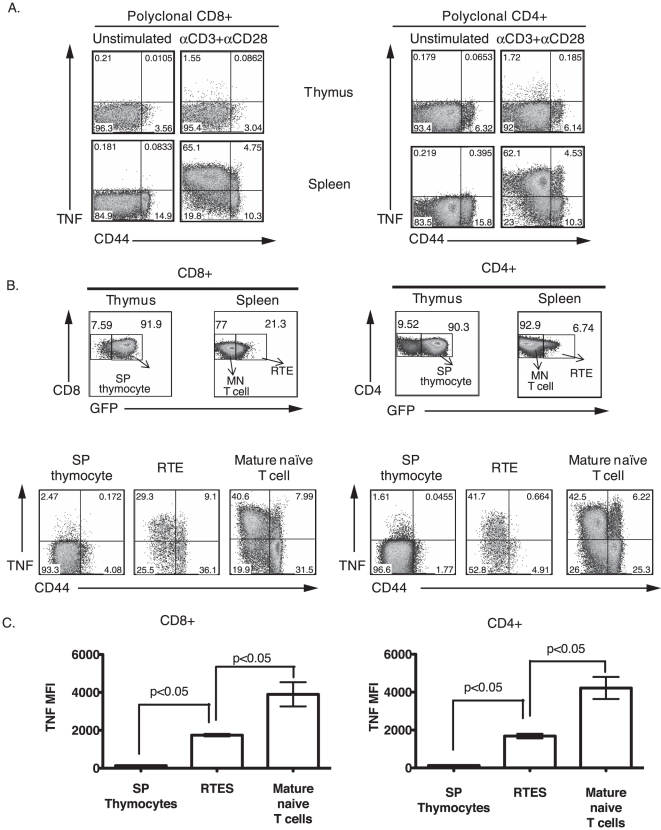
Post-thymic maturation status of naïve polyclonal T cells determines their TNF producing capability. A, The percentages of non-transgenic CD8^+^ and CD4^+^ cells (both CD44^lo^ and CD44^hi^) from thymi and spleens of B6 mice staining positive for TNF cytokine are shown. B, Thymocytes and splenocytes from NG-BAC transgenic mice were stimulated with αCD3+αCD28 for 4 hours and then stained for maturation markers and intracellular TNF. The GFP profile of SP thymocytes, RTEs and MN T cells in the CD8^+^ and CD4^+^ compartments is shown. B and C, The percentages of CD44^lo^ TNF producing cells in the 3 different T cell subsets and their respective average MFI for TNF expression are shown. The average MFI of TNF expression were analysed by one-way ANOVA with a Tukey post-test. The data are representative of 4 individual mice. The error bars indicate SD.

Polyclonal naïve CD4^+^ and CD8^+^ T lymphocytes (CD44^lo^) from secondary lymphoid organs rapidly produce TNF after TCR engagement before gaining other effector functions [8]. However, it is not known if polyclonal SP thymocytes also lack the capability to produce TNF like their transgenic counterparts. To determine this, thymocytes and splenocytes from naïve non-transgenic B6 mice were stimulated using both monoclonal αCD3 and αCD28 antibodies for 4 hrs in vitro, respectively. Fig. 6A shows that, similarly to the transgenic T cells, a lower proportion of polyclonal CD4^+^ CD8^−^ and CD4^−^ CD8^+^ SP thymocytes produced TNF when compared to naïve (CD44^lo^) splenic T cells during TCR stimulation (Fig 6A). This inability to produce TNF was not overcome by increasing the concentrations of the peptide or αCD3 (data not shown). Given this difference and the ability of transgenic SP thymocytes to gradually gain the capability to produce TNF with time in the periphery (shown in Fig 5), we wanted to directly test the ability of polyclonal RTEs that are naturally seeding into the periphery for their ability to produce TNF upon stimulation. For this, we used mice expressing GFP under the control of the Rag2 promoter (NG-BAC transgenic mice). The level of GFP expression by T cells in the periphery of these mice can be used to identify T cells at different stages of post-thymic maturation. GFP^hi^ T cells have resided in the periphery for 0–7 days, GFP^lo^ T cells have resided in the periphery for 7–14 days and GFP^neg^ T cells have joined the MN T cell pool (>14 days in the periphery) [3]. We compared three T cell subsets: SP thymocytes (GFP^hi^), RTEs (GFP^hi+lo^) in the spleen, and MN T cells (GFP^neg^) in the spleen (Fig.6B). A higher proportion of CD8^+^ and CD4^+^ RTEs produced TNF in response to αCD3 and αCD28 stimulation when compared to SP thymocytes (Fig.6B). However, the proportion of CD8^+^ RTEs producing TNF was lower than MN CD8^+^ T cell populations (Fig.6B). This hierarchical pattern of TNF production was also observed on a per cell basis in the three T cell subsets (Fig.6C). In contrast to the CD8^+^ T cell compartment, a similar frequency of CD4^+^ RTE and MN T cells produced TNF, but the MFI of the TNF signal was significantly higher in the CD4^+^ MN T cells relative to both CD4^+^ RTEs and SP CD4^+^ thymocytes (Fig. 6B and 6C). Together these results support our data from the adoptive transfer model indicating that post-thymic maturation confers the complete licensing of naïve T cells to rapidly produce TNF after TCR engagement.

## Discussion

TNF is the earliest known cytokine produced by naïve T cells in secondary lymphoid organs after TCR stimulation [8]. Here, we show that the unique capability of naïve T cells to produce TNF is only acquired via a gradual licensing process that is initiated in the thymus but is completed progressively in the periphery. Our data show that SP thymocytes are functionally less competent to produce TNF upon TCR stimulation relative to naïve T cells in the secondary lymphoid organs. This reduced capability for TNF production is evident at the transcriptional level in SP thymocytes relative to naïve splenic T cells during TCR stimulation. Despite this functional difference, SP thymocytes did not possess any apparent phenotypic defects when compared to naïve splenic T cells during activation (upregulation of CD25 and CD69 and down-regulation of CD62L). The poor ability of SP thymocytes to produce TNF was not overcome upon receiving optimal signals from APCs of secondary lymphoid organs (spleen and lymph node) during TCR activation, suggesting that SP thymocytes possess an intrinsic defect in their ability to produce TNF efficiently upon stimulation. SP thymocytes eventually gain full competence to produce TNF upon TCR stimulation as they undergo post-thymic maturation in the periphery and join the mature-naïve T cell pool in secondary lymphoid organs and this licensing for TNF production does not require homeostatic cell division (data not shown).

Several studies have shown that TNF is expressed in the thymus and have demonstrated that this TNF has a physiological role within the thymus. In situ hybridization studies revealed the localization of TNF mRNA to the cortical regions of the thymus during ontogeny, and studies by Giroir et.al showed that there was constitutive expression of TNF in thymic lymphocytes [52], [53]. In vitro functional studies showed that TNF induced CD25 expression in developing (CD117^+^ CD25^−^) thymocytes in vitro [54]. TNF also induced apoptosis of CD4^−^ CD8^−^ double negative thymocytes that coexpressed both TNFR1 and TNFR2 at low doses but stimulated proliferation at higher doses [55]. Interestingly, TNFR1/2 double KO mice exhibited thymic hypertrophy with an overall increase in total thymocytes but had a normal distribution of SP CD4^+^ and SP CD8^+^ T cell subsets due to absence of apoptosis in DN thymocytes [55]. Collectively, the dual role of TNF in the thymus appears paradoxical and may depend on the location and the quantity of its production at various stages of development.

Alternatively, there is also evidence showing the dangerous effects of deregulated production of TNF in the thymus. For instance, mice that over-express human TNF within the thymus exhibit thymic atrophy, which is primarily associated with premature apoptosis of double negative (DN2) developing thymocytes and diminished numbers of cortical thymic epithelial cells (cTECs) [56]. Mice infected with *Trypanosoma Cruzi* show severe thymocyte depletion of CD4^+^ CD8^+^ DP thymocytes due to an exacerbated inflammatory reaction mediated by TNF [57]. Increased levels of TNF and IFN-γ message have been associated with increased thymocyte deletion and cortical depletion observed in the thymi of patients with Down-Syndrome (DS) [58], [59]. It is proposed that this abnormality may be due to improper interactions between developing thymocytes and thymic stromal cells mediated by elevated levels of LFA-1 and ICAM-1 and an abnormal distribution of ICAM-1 in DS thymi that is then exacerbated by the expression of TNF and IFN-γ in DS thymi [60]. These reports suggest that overproduction of TNF in the thymus may be detrimental to the T cell developmental process. We detected that resting unstimulated SP thymocytes expressed a small level of mature TNF message similar to their splenic counterparts. However, there was no spontaneous production of TNF protein detected in these cells. This suggests that despite having a similar basal level of TNF transcription, there is a lack of translation of the TNF protein under resting conditions in both these subsets. Therefore, the reduced ability of SP thymocytes to rapidly produce TNF during TCR engagement in the thymus under normal circumstances may be beneficial for the survival of SP thymocytes during T cell selection.

A previous report revealed that TNF production by T cells does not require de novo mRNA expression from the TNF locus, as primary CD4 T cells contain a premature TNF transcript which, following TCR engagement, is spliced to form a mature TNF message, resulting in the synthesis of TNF protein in the absence of new transcription [61]. However, we have previously observed that treatment of naïve CD8^+^ T cells with the transcriptional inhibitor actinomycin-D completely abrogated the production of TNF, suggesting that transcription is vital for TNF protein synthesis by naïve CD8^+^ T cells [8]. Our results here show that there is a slight but significant upregulation of TNF message in the CD8^+^ SP thymocytes upon TCR stimulation, but this is insufficient for the optimal production of protein. The splenic T cells, on the other hand, show a dramatic upregulation in TNF message and protein upon activation. Work done in Jurkat T cells and macrophages has revealed that the AU rich regions of the 3′UTR of TNF mRNA is vital for TNF regulation [62]. These findings suggest that there may either be a distinct transcriptional or post-transcriptional control of TNF gene expression in SP thymocytes relative to splenic T cells

Stimulation in the presence of splenocytes partially enabled SP thymocytes to produce TNF efficiently as there was only a partial increase in the proportion of cells producing the cytokine. This effect may be either due to better antigen presentation of exogenous peptides by splenocytes to SP thymocytes. For example, secondary lymphoid cells (spleen and lymph nodes) might offer additional accessory signals leading to enhanced clustering of adhesion molecules during immunological synapse formation and to the partial production of TNF [63], [64]. The alternative possibility is that there are more antigen presenting cells in the splenic environment that increase the strength of TCR-MHC interactions making SP thymocytes more sensitive to stimulation. We are currently exploring these possibilities. Moreover, this effect occurs regardless of whether SP thymocytes are stimulated in the presence of live or killed (γ-irradiated) splenocytes, suggesting that the peptide-MHC complexes and other potential molecules present on the surface of splenocytes are sufficient for this process. Nonetheless, thymocytes are still incapable of producing producing TNF efficiently despite receiving optimal antigen presentation in the splenic environment.

Contact with secondary lymphoid organs is vital for the completion of post-thymic maturation of developing SP thymocytes [1]. RTEs that reach the periphery undergo phenotypic and functional maturation as they become resident in secondary lymphoid organs [3], [65]. Our findings indicate that the full competence for TNF production by naïve T cells in the peripheral T cell pool is acquired gradually in a post-thymic maturation dependent manner. One caveat for the adoptive transfer model is that the recovery of thymocytes from the spleens of the recipient mice decreased over time. Therefore, we cannot exclude the possibility that the increase in the percentage of TNF producing donor CD45.1^+^ SP P14-CD8^+^ thymocytes with time in the periphery may be due to preferential survival of SP thymocytes that are capable of TNF production. Nevertheless, the hierarchical pattern observed in the MFI of the TNF signal of transferred thymocytes and polyclonal RTEs clearly indicates that the progressive gain in the TNF producing capability of naïve T cells occurs as they mature in the periphery.

In mice, developing thymocytes emigrate and populate the periphery at the rate of 1–2% of thymocytes per day throughout the life [66], [67]. Therefore, at any given time in an adult immune system, the naïve T cell pool is comprised of cells at various stages of post-thymic development, unlike neonates whose peripheral lymphoid organs are predominantly populated with RTEs [2], [65]. The post-thymic maturation status of T cells is a component that has been recently shown to influence T cell fate decisions at the time of antigen encounter [2]. This study showed that RTEs produced fewer memory-precursor effector cells (MPECs) and more short-lived effector cells (SLECS) during the immune response to LCMV. Our data show that RTEs produce less TNF relative to MN T cells. Given the immunoregulatory functions of TNF, we speculate that the differential ability of TNF production linked to the post-thymic maturation status of antigen-specific naïve T cells, may also contribute to influencing the fate of the responding T cells during the initial phase of activation.

In conclusion, our findings indicate that the licensing of naïve T cells for rapid TNF production is determined by their developmental state. It is an intrinsic property of the developing T cells that is acquired gradually, where functional maturation in secondary lymphoid organs drives developing naïve T cells to eventually attain full competence to produce TNF efficiently during TCR stimulation.

## Materials and Methods

### Ethics Statement

All the experiments with animals were done in compliance with the institutional guidelines as approved by the University of Massachusetts Institutional Animal Care and Use Committee (IACUC). Animals were maintained in accordance with the *Guide for the Care and Use of Laboratory Animals* (Institute of Laboratory Animal Resources, 1996).

### Mice

Male and female CD45.2^+^ C57BL/6J (B6) mice were purchased from The Jackson Laboratory (Bar Harbor, ME) and used at 6–12 weeks of age. CD45.1^+^ P14 CD8^+^ TCR-transgenic mice, with T cells that recognize the D^b^-restricted, LCMV epitope GP33-41 [68], [69], and CD45.1^+^ SMARTA CD4^+^ TCR-transgenic mice, with T cells that recognize the IA^b^-restricted, LCMV epitope GP61-80 [70], were bred at the University of Massachusetts Medical School (UMMS) Department of Animal Medicine. CD45.1^+^ OT-1 CD8 TCR-transgenic mice, with T cells that recognize the K^b^-restricted, ovalbumin epitope OVA257-264 [71], and CD45.1^+^ OT-2 CD4 TCR-transgenic mice, with T cells that recognize the IA^b^-restricted, ovalbumin epitope OVA323-339 [72] were provided by Dr. Kenneth Rock (Department of Pathology, UMMS, Worcester, MA). NG-BAC transgenic mice, originally obtained from Dr. Michel Nussenzweig were backcrossed to the CD45.1^+^ and CD45.2^+^ background and were used at 6–12 weeks of age [3], [73]. Homozygous C57BL/6Ji-D^btm1^ N12 (H-2D^b^ KO) mice were purchased from Taconic Farms and used at 6 weeks of age. All animals were housed and maintained within the Department of Animal Medicine at UMMS.

### Viruses

Stocks of LCMV, strain Armstrong, and a LCMV variant GP1V virus that possesses an amino acid mutation at position 38 (F to L) in the GP33-41 epitope of LCMV Armstrong were used. This mutation results in the escape of the virus from recognition by LCMV specific D^b^-restricted CTL [74]. Both LCMV stocks were prepared in baby hamster kidney cells (BHK21), as previously described, and mice were infected with 5×10^4^ PFU of each virus strain i.p. [8].

### Flow cytometry and intracellular cytokine assays

Single cell suspensions of thymocytes and splenocytes were prepared in RPMI 1640 supplemented with 10% FBS, 100U/ml penicillin, 100µg/ml streptomycin sulfate and 2mM L-glutamine and stimulated as indicated. For intracellular cytokine assays, lymphocytes (2×10^6^ cells) were stimulated with either 1 µM of the indicated peptide with monoclonal antibodies specific for CD3e (0.25 µg/ml, 145-2C11, BD Pharmingen) and CD28 (2.5µg/ml, 37.51, BD Pharmingen) or with PMA (0.5 µg/ml) and ionomycin (0.5µg/ml) in the presence of GolgiPlug™ (0.1 µg/ml) for 4 hours at 37°C in 5% CO_2_. In some experiments, thymocytes were co-cultured at 1∶1 ratios with either splenocytes from the indicated mouse strains or with the indicated cell populations derived from the spleens of congenic B6 mice and stimulated simultaneously. After the incubation, cells were stained with monoclonal antibodies specific for congenic markers (CD45.1: A20) and (CD45.2: 104), CD4 (RM4-5), CD8 (53-6.7), CD25 (PC61), CD44 (IM7), CD62L (MEL-14), CD69 (H1.2F3), CD24 (M1/69), Qa2 (1-1-2), TCR Vα2 mAb (B20.1) and Vβ8.1 mAb (MR5-2) purchased from BD Pharmingen and CD45RB (C363.16A) from eBioscience. Following the surface stain, cells were fixed and permeabilized using BD Cytofix/Cytoperm™ solution and then stained for intracellular TNF (MP6-XT22 from BD Pharmingen) and CD4 as described previously [8]. For analysis of lymphocytes from NG-BAC transgenic mice, GFP positive cells were determined on the basis of the fluorescence intensity found in SP thymocytes [3]. Fixation slightly diminished the GFP signal during intracellular staining but lymphocytes could still be differentiated as GFP^hi+lo^ and GFP^neg^ cells in the thymus and the spleen. Samples were analyzed using a Becton Dickinson LSRII Flow Cytometer (BD Biosciences) and FlowJo software (Tree star Inc, Ashland, OR).

### Cell purification and enrichment

Single cell suspensions of thymocytes and splenocytes from P14 TCR transgenic mice were purified by staining with anti-CD4, anti-CD8 and anti-CD44 antibodies in 1× PBS with 2% FBS, 2mM EDTA and sorted for CD4^−^ CD8^+^ SP thymocytes and naïve (CD44^low^) splenic T cells using the MoFlo™ XDP cell sorter (Beckton Coulter). The purity of SP CD8 thymocytes was 98% and the purity of CD8^+^CD44^low^ splenic T cells was 90.3%. For cell enrichment, subsets of P14-CD8^+^ T cells were obtained by negative magnetic selection in 1× PBS with 2% FBS, 2mM EDTA. For this, thymocytes were depleted of CD4^+^ cells and splenocytes were depleted of CD4^+^ and CD19^+^ cells by initially staining the cells with biotinylated anti-CD4 (RM4-5; BD Pharmingen) and anti-CD19 (ID3; BD Pharmingen) followed by selection with Streptavidin (SA) microbeads (Miltenyi Biotech, Auburn, CA). The purification of CD8^+^ cells after negative selection was 60% from both tissues. To isolate cell subsets from the CD45.2^+^ splenocytes for the co-culture experiments described above, B cells were positively selected using anti-CD19 microbeads and T cells were positively selected by Thy1.2 microbeads (Miltenyi Biotech). The cells remaining in the flow-through were used as a source of splenic APCs (20% CD11c^+^).

### Real-time PCR

T cell subsets purified either by sorting or enrichment were used as indicated. Total RNA was isolated using a RNA isolation kit (Qiagen Valencia, CA). An additional step was incorporated to remove genomic DNA using a RNAse-free DNAse kit (Qiagen). The concentration of recovered RNA was determined using the NanoDrop® ND-1000 spectrophotometer (Thermo Scientific Willmington, DE). RNA (25 or 50 ng as indicated) was reverse-transcribed into cDNA using Superscript™ III first strand synthesis system (Invitrogen Carlsbad,CA) using oligo(dT) primers. Amplification of the cDNA was then performed by Real time PCR with the SYBR® green mastermix (Applied Biosystems Foster City, CA) using MyiQ™ BioRad icycler. The following TNF primers: FW 5′-CAT CTT CTC AAA ATT CGA GTG ACA A-3′, RV 5′- TGG GAG TAG ACA AGG TAC AAC CC-3′ primers (annealing temp: 60°C and 175 bp product) [75]; β actin primers: FW 5′-CGA GGC CCA GAG CAA GAG AG-3′, RV 5′- CGG TTGGCC TTA GGGTTC AG-3′ and (annealing temp: 62°C and 150 bp product) were used. The following program was used for the real time PCR reaction, Cycle 1: (1×) step 1: 95°C for 10:00; Cycle 2: (40×) step 1: 95°C for 00:15; step 2: 60°C for 1:00; Cycle 3: (1×) step 1: 95°C for 1:00; Cycle 4: (80×) Step 1: 55°C for 00:10. For absolute quantification of the data, standard curves were generated using serial dilution of pCR® 4 –TOPO M13 plasmids containing cDNA clones of TNF and β actin.

### *In-vivo* Brefeldin A (bfA) Assay

This assay was modified from a previously published protocol [45] and used to detect TCR-transgenic T cells producing TNF in vivo. Briefly, unpurified thymocytes or splenocytes from CD45.1^+^ P14 and CD45.1^+^ SMARTA mice 10×10^6^ each (mixed at a 1∶1 ratio) were treated in vitro with 0.5 µg/ml GolgiPlug (BD biosciences) for 20 min at 37°C. Following the incubation, the cells were adoptively transferred into CD45.2^+^ B6 hosts that were infected 2 days previously with 5×10^4^ PFU of LCMV Armstrong or GP1V CTL escape variant. Additionally each mouse received 250 µg of bfA (Sigma) i.v. Four hours after transfer, host spleens were harvested and donor T cells were stained directly for TNF using the intracellular cytokine staining protocol as described above. Additionally, 20×10^6^ P14 thymocytes and splenocytes were transferred separately into uninfected CD45.2^+^ B6 hosts in the absence of bfA. Host spleens were harvested 1, 2, 7 and 14 days after transfer and stimulated in vitro with 1 µM GP33 peptide and αCD28 (2.5µg/ml) in the presence of GolgiPlug™ (0.1 µg/ml) for 4 hours at 37°C in 5% CO_2_ followed by standard intracellular cytokine staining protocol for TNF by donor T cells as described above. Dead cells were excluded using Live Dead Aqua Dead cell stain (Invitrogen; Molecular probes Carlsbad, CA) for this experiment.

### Statistics

Sample analyses were done using Graph Pad Prism (Graph Pad Software). A one-way ANOVA with a Tukey post-test was used to compare multiple samples, with a P value of <0.05 considered significant.

## References

[1] Houston EG, Nechanitzky R, Fink PJ (2008). Cutting edge: Contact with secondary lymphoid organs drives postthymic T cell maturation.. J Immunol.

[2] Makaroff LE, Hendricks DW, Niec RE, Fink PJ (2009). Postthymic maturation influences the CD8 T cell response to antigen.. Proc Natl Acad Sci USA.

[3] Boursalian TE, Golob J, Soper DM, Cooper CJ, Fink PJ (2004). Continued maturation of thymic emigrants in the periphery.. Nat Immunol.

[4] Surh CD, Sprent J (2000). Homeostatic T cell proliferation: how far can T cells be activated to self-ligands?. J Exp Med.

[5] Kaech SM, Ahmed R (2001). Memory CD8+ T cell differentiation: initial antigen encounter triggers a developmental program in naïve cells.. Nat Immunol.

[6] Ansel KM, Lee DU, Rao A (2003). An epigenetic view of helper T cell differentiation.. Nat Immunol.

[7] Joshi NS, Kaech SM (2008). Effector CD8 T cell development: a balancing act between memory cell potential and terminal differentiation.. J Immunol.

[8] Brehm MA, Daniels KA, Welsh RM (2005). Rapid production of TNF-alpha following TCR engagement of naive CD8 T cells.. J Immunol.

[9] Brehm MA, Mangada J, Markees TG, Pearson T, Daniels KA (2007). Rapid quantification of naive alloreactive T cells by TNF-alpha production and correlation with allograft rejection in mice.. Blood.

[10] Mercado R, Vijh S, Allen SE, Kerksiek K, Pilip IM (2000). Early programming of T cell populations responding to bacterial infection.. J Immunol.

[11] Wong P, Pamer EG (2001). Cutting edge: antigen-independent CD8 T cell proliferation.. J Immunol.

[12] van Stipdonk MJ, Lemmens EE, Schoenberger SP (2001). Naïve CTLs require a single brief period of antigenic stimulation for clonal expansion and differentiation.. Nat Immunol.

[13] Tuma RA, Pamer EG (2002). Homeostasis of naïve, effector and memory CD8 T cells.. Curr Opin Immunol.

[14] van Stipdonk MJB, Hardenberg G, Bijker MS, Lemmens EE, Droin NM (2003). Dynamic programming of CD8+ T lymphocyte responses.. Nat Immunol.

[15] Badovinac VP, Harty JT (2006). Programming, demarcating, and manipulating CD8+ T-cell memory.. Immunol Rev.

[16] Pham N-LL, Badovinac VP, Harty JT (2009). A default pathway of memory CD8 T cell differentiation after dendritic cell immunization is deflected by encounter with inflammatory cytokines during antigen-driven proliferation.. J Immunol.

[17] Clark J, Vagenas P, Panesar M, Cope A (2005). What does tumour necrosis factor excess do to the immune system long term?. British Medical Journal.

[18] Smyth MJ, Johnstone RW (2000). Role of TNF in lymphocyte-mediated cytotoxicity.. Microsc Res Tech.

[19] Wajant H, Pfizenmaier K, Scheurich P (2003). Tumor necrosis factor signaling.. Cell Death Differ.

[20] Zheng Y, Saftig P, Hartmann D, Blobel C (2004). Evaluation of the contribution of different ADAMs to tumor necrosis factor alpha (TNFalpha) shedding and of the function of the TNFalpha ectodomain in ensuring selective stimulated shedding by the TNFalpha convertase (TACE/ADAM17).. J Biol Chem.

[21] Kollias G, Douni E, Kassiotis G (1999). The function of tumour necrosis factor and receptors in models of multi-organ inflammation, rheumatoid arthritis,multiple sclerosis and inflammatory bowel disease.. British Medical Journal.

[22] Aggarwal BB (2003). Signalling pathways of the TNF superfamily: a double-edged sword.. Nat Rev Immunol.

[23] Calzascia T, Pellegrini M, Hall H, Sabbagh L, Ono N (2007). TNF-alpha is critical for antitumor but not antiviral T cell immunity in mice.. J Clin Invest.

[24] Pang L, Wang L, Suo T, Hao H, Fang X (2008). Tumor necrosis factor-alpha blockade leads to decreased peripheral T cell reactivity and increased dendritic cell number in peripheral blood of patients with ankylosing spondylitis.. J Rheumatol.

[25] Gunnlaugsdottir B, Skaftadottir I, Ludviksson BR (2008). Naive human T-cells become non-responsive towards anti-TNFalpha (infliximab) treatment in vitro if co-stimulated through CD28.. Scand J Immunol.

[26] Notley CA, Inglis JJ, Alzabin S, McCann FE, McNamee KE (2008). Blockade of tumor necrosis factor in collagen-induced arthritis reveals a novel immunoregulatory pathway for Th1 and Th17 cells.. J Exp Med.

[27] Tracey D, Klareskog L, Sasso EH, Salfeld JG, Tak PP (2008). Tumor necrosis factor antagonist mechanisms of action: a comprehensive review.. Pharmacol Ther.

[28] Bruns H, Meinken C, Schauenberg P, Härter G, Kern P (2009). Anti-TNF immunotherapy reduces CD8+ T cell-mediated antimicrobial activity against Mycobacterium tuberculosis in humans.. J Clin Invest.

[29] Grivennikov SI, Tumanov AV, Liepinsh DJ, Kruglov AA, Marakusha BI (2005). Distinct and nonredundant in vivo functions of TNF produced by t cells and macrophages/neutrophils: protective and deleterious effects.. Immunity.

[30] Kasahara S, Ando K, Saito K, Sekikawa K, Ito H (2003). Lack of tumor necrosis factor alpha induces impaired proliferation of hepatitis B virus-specific cytotoxic T lymphocytes.. J Virol.

[31] Elkon KB, Liu CC, Gall JG, Trevejo J, Marino MW (1997). Tumor necrosis factor alpha plays a central role in immune-mediated clearance of adenoviral vectors.. Proc Natl Acad Sci USA.

[32] Trevejo JM, Marino MW, Philpott N, Josien R, Richards EC (2001). TNF-alpha -dependent maturation of local dendritic cells is critical for activating the adaptive immune response to virus infection.. Proc Natl Acad Sci USA.

[33] Kim EY, Teh H-S (2004). Critical role of TNF receptor type-2 (p75) as a costimulator for IL-2 induction and T cell survival: a functional link to CD28.. J Immunol.

[34] Kim EY, Priatel JJ, Teh S-J, Teh H-S (2006). TNF receptor type 2 (p75) functions as a costimulator for antigen-driven T cell responses in vivo.. J Immunol.

[35] McKarns SC, Schwartz RH (2008). Biphasic regulation of Il2 transcription in CD4+ T cells: roles for TNF-alpha receptor signaling and chromatin structure.. J Immunol.

[36] Kim EY, Teh S-J, Yang J, Chow MT, Teh H-S (2009). TNFR2-deficient memory CD8 T cells provide superior protection against tumor cell growth.. J Immunol.

[37] Suresh M, Singh A, Fischer C (2005). Role of tumor necrosis factor receptors in regulating CD8 T-cell responses during acute lymphocytic choriomeningitis virus infection.. J Virol.

[38] Singh A, Wüthrich M, Klein B, Suresh M (2007). Indirect regulation of CD4 T-cell responses by tumor necrosis factor receptors in an acute viral infection.. J Virol.

[39] Singh A, Suresh M (2007). A role for TNF in limiting the duration of CTL effector phase and magnitude of CD8 T cell memory.. J Leukoc Biol.

[40] Schodin BA, Tsomides TJ, Kranz DM (1996). Correlation between the number of T cell receptors required for T cell activation and TCR-ligand affinity.. Immunity.

[41] Ashton-Rickardt PG, Bandeira A, Delaney JR, Van Kaer L, Pircher HP (1994). Evidence for a differential avidity model of T cell selection in the thymus.. Cell.

[42] Schott E, Bertho N, Ge Q, Maurice MM, Ploegh HL (2002). Class I negative CD8 T cells reveal the confounding role of peptide-transfer onto CD8 T cells stimulated with soluble H2-Kb molecules.. Proc Natl Acad Sci USA.

[43] Davey GM, Schober SL, Endrizzi BT, Dutcher AK, Jameson SC (1998). Preselection thymocytes are more sensitive to T cell receptor stimulation than mature T cells.. J Exp Med.

[44] Proietto AI, Lahoud MH, Wu L (2008). Distinct functional capacities of mouse thymic and splenic dendritic cell populations.. Immunol Cell Biol.

[45] Liu F, Whitton JL (2005). Cutting edge: re-evaluating the in vivo cytokine responses of CD8+ T cells during primary and secondary viral infections.. J Immunol.

[46] McCaughtry TM, Wilken MS, Hogquist KA (2007). Thymic emigration revisited.. J Exp Med.

[47] Crispe IN, Bevan MJ (1987). Expression and functional significance of the J11d marker on mouse thymocytes.. J Immunol.

[48] Vernachio J, Li M, Donnenberg AD, Soloski MJ (1989). Qa-2 expression in the adult murine thymus. A unique marker for a mature thymic subset.. J Immunol.

[49] Gabor MJ, Godfrey DI, Scollay R (1997). Recent thymic emigrants are distinct from most medullary thymocytes.. Eur J Immunol.

[50] Uldrich AP, Berzins SP, Malin MA, Bouillet P, Strasser A (2006). Antigen challenge inhibits thymic emigration.. J Immunol.

[51] Chen W (2004). The late stage of T cell development within mouse thymus.. Cell Mol Immunol.

[52] Deman J, Martin MT, Delvenne P, Humblet C, Boniver J (1992). Analysis by in situ hybridization of cells expressing mRNA for tumor-necrosis factor in the developing thymus of mice.. Developmental Immunology.

[53] Giroir BP, Brown T, Beutler B (1992). Constitutive synthesis of tumor necrosis factor in the thymus.. Proc Natl Acad Sci USA.

[54] Zúñiga-Pflücker JC, Jiang D, Lenardo MJ (1995). Requirement for TNF-alpha and IL-1 alpha in fetal thymocyte commitment and differentiation.. Science.

[55] Baseta JG, Stutman O (2000). TNF regulates thymocyte production by apoptosis and proliferation of the triple negative (CD3-CD4-CD8-) subset.. J Immunol.

[56] Liepinsh DJ, Kruglov AA, Galimov AR, Shakhov AN, Shebzukhov YV (2009). Accelerated thymic atrophy as a result of elevated homeostatic expression of the genes encoded by the TNF/lymphotoxin cytokine locus.. Eur J Immunol.

[57] Pérez AR, Roggero E, Nicora A, Palazzi J, Besedovsky HO (2007). Thymus atrophy during Trypanosoma cruzi infection is caused by an immuno-endocrine imbalance.. Brain Behav Immun.

[58] Murphy M, Epstein LB (1992). Down syndrome (DS) peripheral blood contains phenotypically mature CD3+TCR alpha, beta+ cells but abnormal proportions of TCR alpha, beta+, TCR gamma, delta+, and CD4+ CD45RA+ cells: evidence for an inefficient release of mature T cells by the DS thymus.. Clin Immunol Immunopathol.

[59] Murphy M, Friend DS, Pike-Nobile L, Epstein LB (1992). Tumor necrosis factor-alpha and IFN-gamma expression in human thymus. Localization and overexpression in Down syndrome (trisomy 21).. J Immunol.

[60] Murphy M, Insoft RM, Pike-Nobile L, Derbin KS, Epstein LB (1993). Overexpression of LFA-1 and ICAM-1 in Down syndrome thymus. Implications for abnormal thymocyte maturation.. J Immunol.

[61] Chang J, Parnes J, Garrison Fathman C (1998). T Cell Receptor (TCR) engagement leads to activation-induced splicing of tumor necrosis factor (TNF) nuclear pre-mRNA.. Journal of Experimental Medicine.

[62] Buxadé M, Parra J, Rousseau S, Shpiro N (2005). The Mnks are novel components in the control of TNFα biosynthesis and phosphorylate and regulate hnRNP A1.. Immunity.

[63] Sims TN, Dustin ML (2002). The immunological synapse: integrins take the stage.. Immunol Rev.

[64] Inaba K, Pack M, Inaba M, Sakuta H, Isdell F (1997). High levels of a major histocompatibility complex II-self peptide complex on dendritic cells from the T cell areas of lymph nodes.. J Exp Med.

[65] Opiela SJ, Koru-Sengul T, Adkins B (2009). Murine neonatal recent thymic emigrants are phenotypically and functionally distinct from adult recent thymic emigrants.. Blood.

[66] Berzins S, Godfrey D, Miller J (1999). A central role for thymic emigrants in peripheral T cell homeostasis.. Proc Natl Acad Sci USA.

[67] Berzins SP, Uldrich AP, Sutherland JS, Gill J, Miller JFAP (2002). Thymic regeneration: teaching an old immune system new tricks.. Trends Mol Med.

[68] Pircher H, Bürki K, Lang R, Hengartner H, Zinkernagel RM (1989). Tolerance induction in double specific T-cell receptor transgenic mice varies with antigen.. Nature.

[69] Pircher H, Moskophidis D, Rohrer U, Bürki K, Hengartner H (1990). Viral escape by selection of cytotoxic T cell-resistant virus variants in vivo.. Nature.

[70] Oxenius A, Bachmann MF, Zinkernagel RM, Hengartner H (1998). Virus-specific MHC-class II-restricted TCR-transgenic mice: effects on humoral and cellular immune responses after viral infection.. Eur J Immunol.

[71] Hogquist KA, Jameson SC, Heath WR, Howard JL, Bevan MJ (1994). T cell receptor antagonist peptides induce positive selection.. Cell.

[72] Robertson J, Jensen P, Evavold B (2000). DO11. 10 and OT-II T cells recognize a C-terminal ovalbumin 323–339 epitope.. The Journal of Immunology.

[73] Yu W, Nagaoka H, Jankovic M, Misulovin Z, Suh H (1999). Continued RAG expression in late stages of B cell development and no apparent re-induction after immunization.. Nature.

[74] Hahn K, Jewell D, Wilson I, Oldstone M (1995). CTL Escape Viral Variants I. Generation and Molecular Characterization.. Virology.

[75] Valckx D, Decallonne B, Bouillon R, Mathieu C (2001). An overview of real-time quantitative PCR: applications to quantify cytokine gene expression.. Methods.

